# Energy Absorption Behavior of Carbon-Fiber-Reinforced Plastic Honeycombs under Low-Velocity Impact Considering Their Ply Characteristics

**DOI:** 10.3390/ma17174257

**Published:** 2024-08-28

**Authors:** Zheng Liu, Kai Zou, Zhendong Zhang

**Affiliations:** School of Mechanical Engineering, Nanjing University of Science and Technology, Nanjing 210094, China; liuzheng@njust.edu.cn

**Keywords:** carbon-fiber-reinforced plastic (CFRP), honeycomb structure, low-velocity impact (LVI), cushioning energy absorption, progressive failure, finite element simulation

## Abstract

Honeycomb structures made of carbon-fiber-reinforced plastic (CFRP) are increasingly used in the aerospace field due to their excellent energy absorption capability. Attention has been paid to CFRP structures in order to accurately simulate their progressive failure behavior and discuss their ply designability. In this study, the preparation process of a CFRP corrugated sheet (half of the honeycomb structure) and a CFRP honeycomb structure was illustrated. The developed finite element method was verified by a quasi-static test, which was then used to predict the low-velocity impact (LVI) behavior of the CFRP honeycomb, and ultimately, the influence of the ply angle and number on energy absorption was discussed. The results show that the developed finite element method (including the user-defined material subroutine VUMAT) can reproduce the progressive failure behavior of the CFRP corrugated sheet under quasi-static compression and also estimate the LVI behavior. The angle and number of plies of the honeycomb structure have an obvious influence on their energy absorption under LVI. Among them, energy absorption increases with the ply number, but the specific energy absorption is basically constant. The velocity drop ratios for the five different ply angles are 79.12%, 68.49%, 66.88%, 66.86%, and 60.02%, respectively. Therefore, the honeycomb structure with [0/90]s ply angle had the best energy absorption effect. The model proposed in this paper has the potential to significantly reduce experimental expenses, while the research findings can provide valuable technical support for design optimization in aerospace vehicle structures.

## 1. Introduction

The honeycomb structure, being a porous structure, possesses attributes such as light weight, sound absorption, heat insulation, and flexible design. Consequently, it can effectively achieve a balance between light weight requirements and high strength demands [1]. As a result of these advantages, it finds extensive applications in sectors including automotive engineering, the marine industry, and aerospace technology [2]. The main materials used in previous studies on honeycomb structures were aluminium honeycomb and Nomex honeycomb, which exhibit the drawbacks of limited thermal stability and low strength, respectively [3,4,5,6,7,8]. With the advancement of aerospace technology, there is an increasing demand for structures with enhanced energy absorption capacity, light weight, and high strength. However, the existing honeycomb materials are unable to meet the requirements of complex environmental adaptation in specialized applications. Therefore, it is imperative to develop novel materials for honeycomb structures and investigate their response under impact loads.

Carbon-fiber-reinforced plastics (CFRPs) are composed of extremely fine carbon fibers (typically between 5 and 10 μm in diameter) and a polymer matrix such as plastics and resins [9]. The carbon fibers themselves have excellent mechanical properties, such as high strength, high modulus, and low density. When embedded in the polymer matrix, they can form a new material with low weight, high strength, corrosion resistance, and fatigue resistance [10]. Research into CFRP began in the 1950s, and its development was driven mainly by aerospace and military requirements [11]. With the increasing demand for light weight and durability, CFRP gradually expanded to the automotive industry, construction, sports equipment, and other fields [12,13,14]. The mechanical research progress of CFRP covers many aspects, such as strength and stiffness optimization, fatigue behavior research, impact response analysis, interfacial shear strength, and so on [15,16,17,18,19,20,21].

Considering the possible impact load of CFRP in practical applications, the impact response has been studied in detail. Fedorenko et al. [22] proposed a modelling method of the crushing behavior of absorbing structural components made of fiber-reinforced composites. The damage rate was used to represent the high-rate behavior, which improveds the accuracy of the analysis. Ge et al. [23] studied the dynamic response of a CFRP laminate under low-velocity impact (LVI) using experimental and numerical methods and established a finite element model (FEM) that can calculate intralaminar and interlaminar damage. This FEM can better predict the twill weave composite response time, impact resistance, damage morphology, and delamination damage of the CFRP laminate. Seamon et al. [24] investigated the damage sequence during the impact of a CFRP structure, and they found that the compression failure of splitting and the delamination of the panel and impact surface can cause a series of damages; they therefore summarized the correlation between impact energy, panel configuration, and damage mode. Li et al. [25] established a multi-scale model to study the low-temperature mechanical properties of CFRP, where the influence of a low-temperature environment on the mechanical properties and failure behavior of CFRP was analyzed. The results showed that the CFRP was characterized by embrittlement transition and delamination damage, and these provide an effective method for structural performance analysis and material design for low-temperature applications. Liu et al. [26] carried out a transverse impact experiment and simulation of carbon–glass hybrid fiber-reinforced polymer composite laminate, where a variety of impact deformations and damage mechanisms were revealed. The results showed that carbon fiber had higher tensile and shear modulus than glass fiber, and as the proportion of carbon fiber increased, the composite had higher impact resistance.

The combination of CFRP and honeycomb is one of the research directions in materials science today. CFRP itself has the characteristics of light weight and high strength, and the honeycomb can effectively increase the strength and stiffness of the structure. Therefore, their combination can further improve the energy absorption properties and also enhance the safety and reliability of the material. The combination of the two can meet the needs of modern engineering for lightweight and high-performance materials [27]. Xu et al. [28] proposed a preparation method for a multi-layer honeycomb sandwich structure with the same volume density, effectively eliminating the influence of bulk density change on the mechanical properties of the experimental specimen. They also summarized the transverse size change rules of the honeycomb sandwich structure and identified the main causes of irregular deformation at the boundary. Feng et al. [29] used FFF (fused filament fabrication) technology to fabricate the panel/core layer integrated sandwich structure, and found that the staggered fiber design can significantly improve the mechanical properties of the sandwich structure (where the elastic modulus improved by 44%, the strength improved by 119%). The main failure modes include fiber pull-out and fracture, delamination, local core layer crushing, and fiber/matrix and panel/core layer debonding. Shunmugesh et al. [30] performed finite element modeling of epoxy resin and honeycomb, and investigated the performance of the designed composites under various tensile loads, which showed good performance under tension and compression. Zhang et al. [31] studied the failure mechanism and process defects of the 3D printing of a continuous CFRP circular honeycomb structure. In their study, they identified the main factor of microscopic pores and the weak interface damage mode, and revealed the different correlations between process defects and failure mechanism in 3D printing, providing guidance for the lightweight and robust design of circular honeycomb composite structures.

To summarize, there is a scarcity of research on CFRP honeycomb structures, and the investigation into its response and damage mechanism under LVI load remains unexplored. The manufacturing process of CFRP honeycomb is complicated, and conducting numerous tests incurs relatively high costs. To comprehensively investigate the response of CFRP honeycomb under impact loads, it is imperative to develop a set of calculation models capable of accurately simulating the testing conditions.

In this study, the CFRP honeycomb structure and its component corrugated sheets are fabricated and subjected to quasi-static compression tests. The CFRP honeycomb structure is accurately modeled using the finite element method, based on the test conditions. The model’s accuracy is validated against experimental results, after which it is employed to simulate the response of CFRP honeycomb under low-speed impact. Furthermore, the factors influencing its energy absorption capacity are discussed. The aforementioned model offers significant cost savings for research purposes and boasts a prolonged lifespan. It serves as a dependable source of technical support for structural optimization design within the aerospace industry, thereby holding substantial academic value and practical significance.

## 2. Materials and Experiments

### 2.1. Material Introduction

The honeycomb structures were fabricated by CFRP prepreg (TORAYCA T700/2510), produced by Weihai GuangWei Composites Co., Ltd. (Weihai, China) The thickness of a single ply prepreg is 0.25 mm. The fiber areal weight is 198 g/m^2^. The material properties of the prepreg are shown in Table 1.

### 2.2. Fabrication Procedure

**Figure 1:**
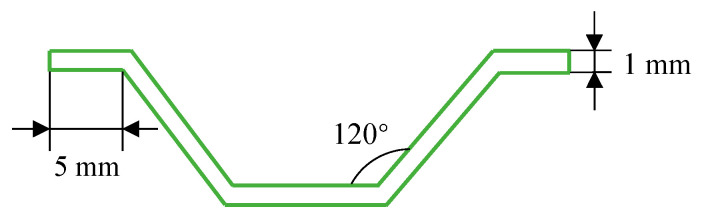
Cross-section of the partial corrugated sheet.

The CFRP honeycomb structures designed in this paper are composed of periodic corrugated sheets through bonding. The cross-section size of the corrugated sheet is shown in Figure 1. The ply mode of the corrugated sheet is [0/90]_s_, which consists of 4 CFRP prepreg layers, with a total thickness of 1 mm and a height of 50 mm. One end of the board is designed with a 45° chamfer so that it can be stably crushed, preventing the middle part from being destroyed first under the impact force and the overall loss of load-bearing capacity.

**Figure 2:**
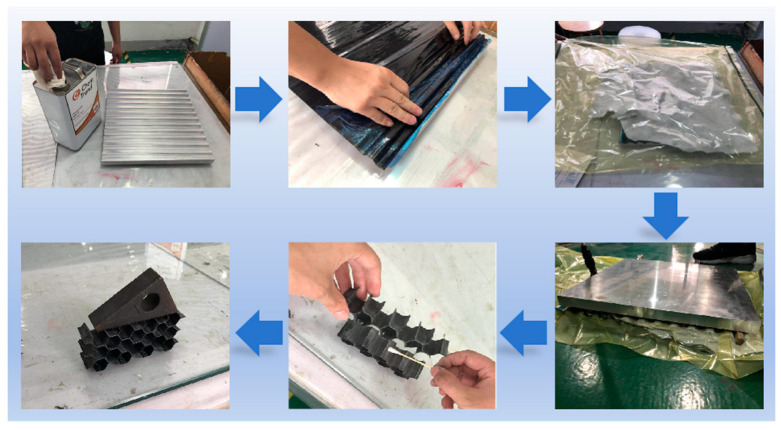
The manufacturing process of CFRP corrugated sheet and honeycomb.

The fabrication procedure of corrugated sheets and honeycomb structures is shown in Figure 2. The specific manufacturing process was as follows. (i) Firstly, the stainless steel molds were cleaned with acetone. The PMR-EZ liquid release agent was evenly applied on the surfaces and edges of the molds. The molds were exposed to air until the surfaces were completely dry. (ii) Secondly, the CFRP prepreg was cut to an appropriate size and tightly compressed against the molds to ensure a precise fit with the concave and convex surfaces. (iii) Then, the molds were enveloped with a layer of felt, followed by being hermetically sealed within a vacuum bag for vacuum treatment. Subsequently, a curing process for the CFRP prepreg was carried out in an autoclave, involving a heating stage at 90 °C for 30 min, followed by an additional heating stage at 125 °C for 60 min. After the molds were cooled down to room temperature, corrugated sheets were obtained by removing the molds. (iv) Finally, the cured corrugated sheets were sectioned into 50 mm wide strips and then bonded together using the same epoxy resin as the prepreg, forming a honeycomb structure. A standard size specimen was obtained after polishing with sandpaper with a grit size of P100.

### 2.3. Quasi-Static Experiments

**Figure 3:**
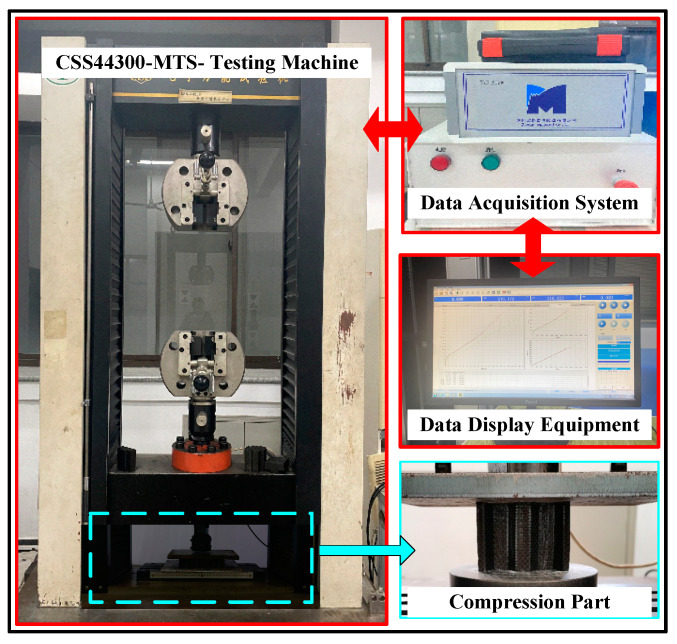
Details of testing machine and experiment.

In the impact test, the CFRP sheet specimen was fixed vertically to the freshly ground, clean, dry steel surface in the test fixture. The specimen is self-supporting, requiring no external support. The rigid head at the upper end can be moved up and down in a direction perpendicular to the rigid plate at the lower end. During the quasi-static collapse, the data acquisition system was used to record the displacement–load curve throughout the experiment and to capture the collapse deformation process of the structural specimen. As the single corrugated sheet structure is unsupported and unfixed, the bottom of the specimen should be polished and flat to prevent instability of the specimen during the experiment, which could affect the authenticity of the collected data. The test machine used this time is the CSS-44300 universal material testing machine manufactured by China Changchun Test Machine Research Institute, as shown in Figure 3, with a capacity of 100 KN. The quasi-static strain rate calculated by Equation (1) is between 0.00025 and 0.0025/s. The axial size of the specimen is 50 mm, and the compression head is set to crush the single corrugated sheet specimen at a velocity of 7.5 mm/min.

(1) $$\dot{\varepsilon }=\frac{v}{L}$$

where $\dot{\varepsilon }$ is the strain rate, v is the compression velocity, L is the original length of the specimen.

**Figure 4:**
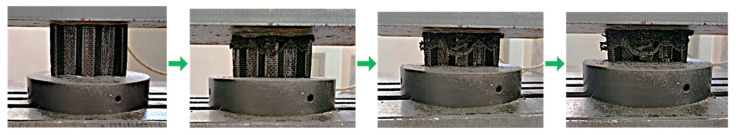
Quasi-static crush process of composite corrugated sheets.

As shown in Figure 4, the single corrugated sheet specimen was stably crushed, where delamination occurred and the broken material was flipped to both sides. In addition, the crushing failure of the matrix and the fracture of the fibers occurred. From the second sub-figure, the corrugated sheet structure gradually assumed an inverted trapezoidal shape, and the freedom of movement of the top end was constrained by the compression head. Since there was no constraint perpendicular to the loading direction, and the cross-section angle of the corrugated sheet structure was 120°, the failed sheet easily opened out on both sides, exhibiting an inverted trapezoidal shape.

## 3. Finite Element Simulation of CFRP

### 3.1. Finite Element Model

**Figure 5:**
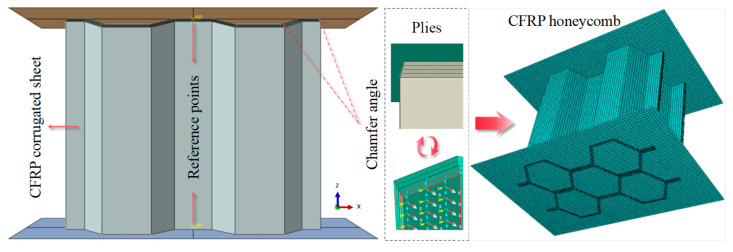
Finite element simulation model of a partial single corrugated sheet.

As shown in Figure 5, the specimen is assembled vertically between the upper and lower rigid panels, and the chamfer is designed for the specimen. As the thickness of the single fiber layer in the specimen is only 0.25 mm, its size is much smaller than 1/10 of the overall structural size. At the same time, the corrugated sheet is subjected to axial load and the thickness direction does not bear the load, so it is advisable to use the shell element for modeling. Therefore, the continuous shell element (SC8R) was used to model both the corrugated sheet and honeycomb structure.

**Figure 6:**
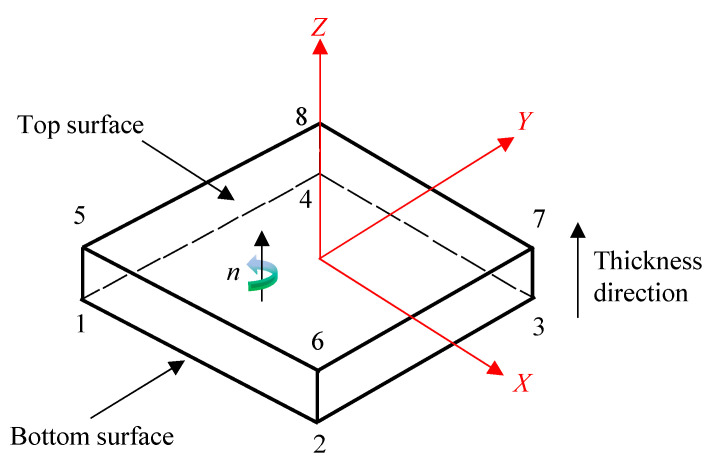
Definition of a continuous shell element, where n is the normal direction of the element.

The continuous shell element in ABAQUS possesses the characteristics of a 3D solid element, and its dynamics and constitutive behavior are similar to those of a traditional shell element. This type of element can accurately simulate multi-layer CFRP and also improve the computational efficiency. Since the continuum shell behaves differently in the thickness direction compared to the in-plane direction, a reasonable orientation is important. By default, the top and bottom of the element, as well as the normal, superposition, and thickness directions, are defined by node connectivity. For the quadrilateral continuous shell element (SC8R), the face formed by nodes 1, 2, 3, and 4 is considered the bottom, and the face formed by nodes 5, 6, 7, and 8 is the top, as shown in Figure 6. Both the stacking direction and the thickness direction are defined as the direction from the bottom surface to the top surface. In ABAQUS, the continuous shell can only be swept in the positive direction of the *Z*-axis of the coordinate system. The finite element model of the rigid plate is set up using the discrete rigid body element (R3D4), and the mesh type is a discrete rigid element.

Fiber-reinforced composites are an anisotropic material, which requires a definition of the material direction. In this paper, the material direction definition is adopted based on the rectangular coordinate system. In the FE model, the fiber direction of the single corrugated sheet is along the height direction (*Z*-direction). However, there are concave and convex parts in the corrugated sheet, all surfaces are not coplanar, and the direction perpendicular to the fiber is not unified in the global coordinate system. Therefore, the local coordinate system is used, where 1 is the fiber direction, 2 is the transverse fiber direction, 3 is the thickness direction, and S is the fiber stacking direction.

In this paper, based on the continuous shell element of the plane stress assumption, for small strain shell elements in ABAQUS 6.14/Explicit, the transverse shear stiffness is determined based on the effective shear modulus. When a user subroutine (VUMAT) is used to define the material response of a shell element, the transverse shear stiffness values cannot be calculated automatically, so the definition of the transverse shear stiffness is required. The definition of the appropriate stiffness depends on the material composition of the shell and its laminate, i.e., how the material is distributed across the thickness of the section. When calculating the transverse shear stiffness, ABAQUS assumes that the shell section direction is the main bending direction (bending about one main direction does not require constrained moments about the other direction). For composite shells with orthogonal anisotropic layers exhibiting asymmetry about the shell mid-plane, the shell section direction may not be the main bending direction. In this case, the transverse shear stiffness is a less accurate approximation. Changes occur when different shell section orientations are used. The transverse shear stiffness is calculated only at the beginning of the analysis, based on the initial elastic properties given in the model data, and any change in transverse shear stiffness due to changes in material stiffness during the analysis is ignored. The transverse shear stiffness is given as the initial linear elastic stiffness corresponding to the pure transverse shear strain in the shell response.

For a homogeneous shell made of a linear orthotropic anisotropic elastic material, the transverse shear stiffness shall be defined [32], i.e.,

(2) $$K_{11}^{ts}=\frac{5}{6}G_{13}t,\;K_{22}^{ts}=\frac{5}{6}G_{23}t,\;K_{12}^{ts}=0$$

where $G_{13}$ and $G_{23}$ are the shear modulus in the out-of-plane direction, and t is the thickness of the orthotropic anisotropic elastic material.

Under the premise of ensuring the simulation accuracy, in order to improve the computational efficiency, the mesh size of the single corrugated sheet is set to 1 mm, with a total of 30,800 meshes, and the thickness direction unit is 1 layer. In this, a 2D constitutive model is adopted, and the VUMAT refers to Refs. [14,33]. The material parameters of TORAYCA^®^ T700/2510 are given in Table 2. The values given by the material parameters can be adjusted within 15% to meet the calculation requirements. It is not comprehensive enough to solely determine whether a material point is completely invalid and thus delete the failing element. Furthermore, to ensure smooth calculation, elements with a high degree of deformation distortion should be deleted. To avoid excessive deformation of some cells leading to premature termination of analysis calculations, failed continuous shell elements were removed from the model.

After much trial work, the element deletion based on damage factor is combined with deletion criteria based on the deformation. Damage-based element deletion is activated when the fiber damage variable, the matrix damage variable, or the shear damage variable reaches the maximum specified value. Here, the principal logarithmic strain in tension or compression, combined with a deformation-based deletion criterion, is used to determine whether its maximum or minimum specified value has been reached. Reference values for strain-based deletion are given in Table 3, and these values can be adjusted within a certain range.

The bonding layer between the carbon fiber layers is almost negligible, which can be considered as a zero-thickness adhesive layer. Therefore, the zero-thickness cohesive element was used to simulate the delamination failure between the layers. In the 3D problem, the continuum-based constitutive model assumes that one direct (through thickness) strain, two transverse shear strains, and all (six) stress components are active at the material point. The model adopts general contact and surface-to-surface contact algorithms to prevent penetration between the specimen and the rigid plate. As noted in Ref. [10], friction during crushing plays an important role in the analysis. According to Ref. [11], the coefficient of friction of the interlayer surface of the debonded material was assumed to be 0.3, and the coefficient of friction between the composite and the rigid plate was set at 0.12. After a delamination occurs between any two adjacent layers, the general contact definition is automatically updated to account for possible contacts. The compression velocity of the quasi-static experiment is 7.5 mm/min. If the motion velocity of the rigid plate is set to the value given in the simulation, the calculation using the ABAQUS/Explicit solver becomes very time-consuming. On the premise of ensuring calculation accuracy, it is necessary to adopt certain technical means to speed up the calculation process. The estimation equation for each incremental time step in the stability limit of the explicit kinetic process is

(3) $$\left\{\begin{array}{c} ∆t=\left(\frac{L^{e}}{c_{d}}\right)\\ c_{d}=\sqrt{\frac{E}{\rho }} \end{array}\right.$$

where $L^{e}$ is the smallest characteristic cell length, $c_{d}$ is the expansion wave velocity of the material, E is the elastic modulus, and ρ is the density of the material.

At the beginning of the analysis, the mass scaling capability was used to obtain reasonable run times [11]. In this simulation, the mass scaling coefficient was set to 1000, i.e., the mass of the CFRP specimen was increased by three orders of magnitude. Based on the above series of analyses, the modeling and simulation were carried out, including the quasi-static collapse simulation of the single corrugated sheet. The accuracy of the numerical model was verified by comparing the collapse morphology and load–displacement curves of the test with those of the simulation, referring to [32].

### 3.2. FEM Validation under Quasi-Static Compression

**Figure 7:**
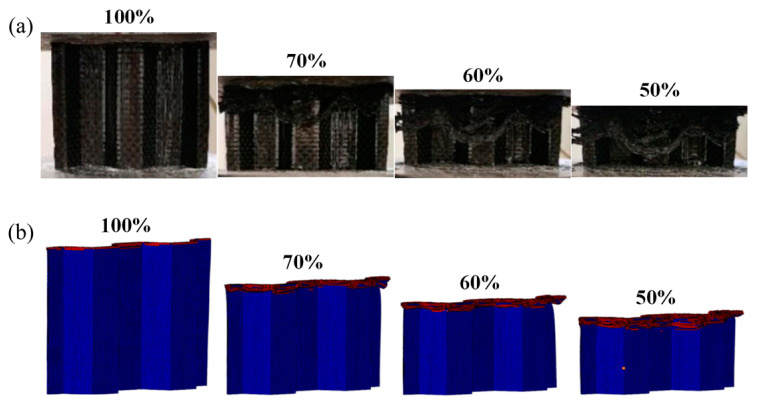
Quasi-static crushing process of a single corrugated sheet. (**a**) Experiments, (**b**) simulations.

**Figure 8:**
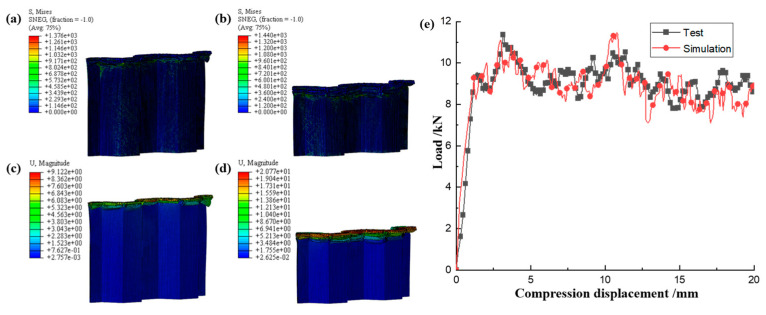
Stress nephogram of (**a**) s = 10 mm and (**b**) s = 20 mm, displacement nephogram of (**c**) s = 10 mm and (**d**) s = 20 mm under LVI, and (**e**) load–displacement curves.

Based on the FEM established above, the quasi-static collapse simulation of a single corrugated sheet was carried out. The accuracy of the FEM was verified by comparing the failure morphology and load–displacement curves of the test and simulation. As shown in Figure 7, the failure modes such as matrix failure, fiber fracture, matrix fragmentation, and layer failure during quasi-static compression of the single corrugated sheet are very close to the experimental phenomena. From the compression behavior in Figure 8, it can be seen that the established FEM has good accuracy in simulating the progressive crushing of CFRP. The load–displacement curves of the experiment and simulation in Figure 8e exhibit a high degree of consistency. The observed slight disparity in the displacement range of 5–10 mm can be attributed to the non-uniformity of the internal structure of the specimen during the crushing process, which introduces a certain level of randomness into the crushing mechanism. Overall, the load fluctuates around a stable value throughout the entire crushing process. Therefore, this FEM can be used to simulate more conditions to study the energy absorption behavior of CFRP under compression load.

### 3.3. FEM Validation under LVI

**Figure 9:**
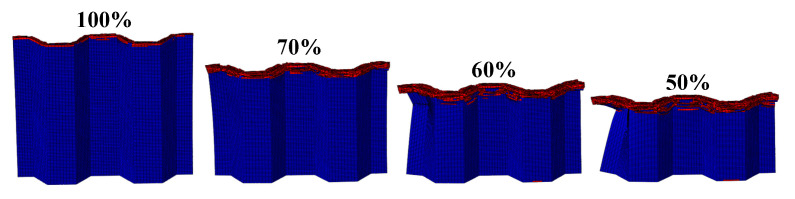
Crush failure results of single corrugated sheet under LVI.

**Figure 10:**
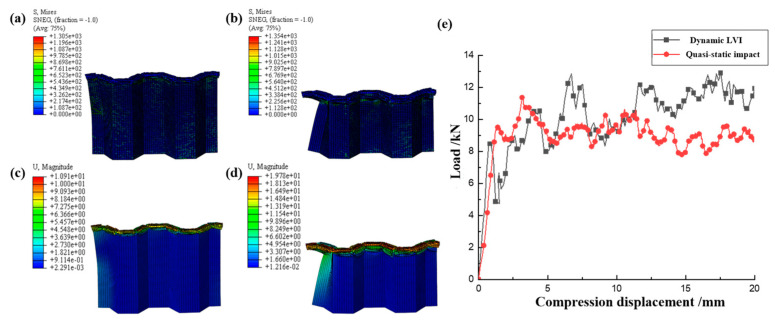
Stress nephogram of (**a**) s = 10 mm and (**b**) s = 20 mm, and displacement nephogram of (**c**) s = 10 mm and (**d**) s = 20 mm under LVI, and (**e**) load–displacement curves.

The FEM of a single CFRP corrugated sheet under LVI is similar to the quasi-static model, except that the impact velocity is set to 5 m/s, resulting in a strain rate of 100 s^−1^. The mechanical properties of CFRP have been shown in numerous studies to exhibit negligible changes in the range of medium to low strain rates [22,23,24,25,26,27,28,29,30]. Therefore, the strain rate effect can be ignored when establishing the LVI model in the study of the energy absorption behavior of CFRP to ensure a more focused analysis. The failure morphology of a single CFRP corrugated sheet under LVI is illustrated in Figure 9. The failure modes observed in the CFRP are delamination failure, fiber fracture, and matrix crushing, which are similar to those observed in Figure 7 during quasi-static crushing. The single CFRP corrugated sheet not only exhibits the same failure mode as under quasi-static compression conditions, but also exhibits local buckling instability under high dynamic impact loading. The poor overall stiffness of the single corrugated laminate indicates its limited ability to withstand impact loads. Therefore, it is necessary to integrate the single corrugated sheet into a honeycomb structure to improve its impact resistance. The stress and deformation nephogram of the uncrushed area of the single corrugated sheet during impact is shown in Figure 10. The elements in the contact area with the rigid plate at the top end are eliminated, and the failed elements do not bear the load. Therefore, the stress values of the meshes at the top are zero, and the stress away from the contact area with the rigid plate is smaller. The stress in the contact area and adjacent area is large because this part is subjected to a large impact load and is gradually crushed with the downward movement of the rigid plate. Overall, the load–displacement curves of quasi-static compression and LVI have the same trend, but the buckling instability in LVI has a low trough after the initial peak, after which the load plateau shows a greater fluctuation than that of quasi-static, which is characteristic of dynamic impact.

## 4. Results and Discussion

### 4.1. LVI Behavior of Honeycomb Structures

#### 4.1.1. LVI Validation of Honeycomb Structures

**Figure 11:**
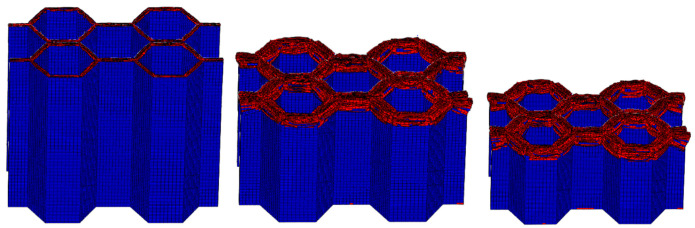
LVI process at 5 m/s where the honeycomb has 4 plies.

**Figure 12:**
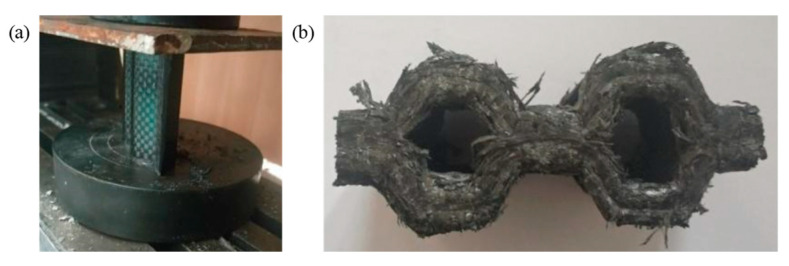
Quasi-static crushing of the simplest honeycomb: (**a**) compression state, (**b**) crushing morphology.

**Figure 13:**
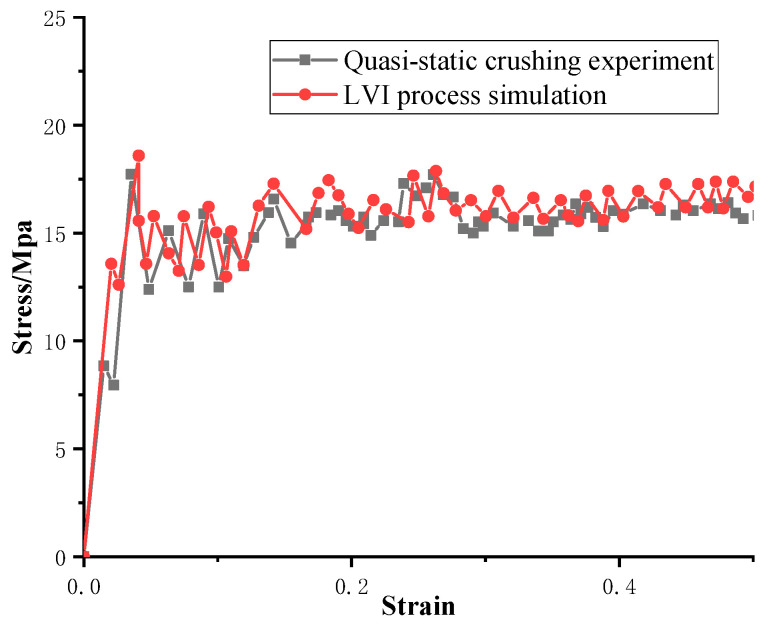
Stress–strain curves of quasi-static crushing experiment and LVI process simulation of honeycomb.

The FE pre-processing of the corrugated sheet under LVI is identical to that for the quasi-static simulation, except for the mass scaling. The impact velocity is set to 5 m/s and the mass of the rigid plate is set to 100 kg to avoid rebound caused by insufficient kinetic energy during the impact. From the above comparative analyses, it can be seen that the influence of the strain rate effect can be reasonably ignored for the LVI in the range of medium to low strain rates. Therefore, the rate effect is not considered in the subsequent LVI simulation. The damage morphology of the CFRP honeycomb formed by the combination of the above four corrugated sheets is shown in Figure 11. The damage pattern is essentially the same as that observed in the quasi-static case, with failure modes including delamination, fiber breakage, and crushed matrix. However, stable progressive damage occurs without instability, which is an advantage of the honeycomb configuration. In conjunction with the quasi-static experimental results of the honeycomb, i.e., Figure 12, it can be observed that the damage morphology is largely consistent with the simulation results under LVI, which are characterized by progressive compression and collapse damage. It is observed that the crushed body flips from the center to the sides, while the crushed matrix and fibers flip to the outside and inside of the honeycomb holes, respectively. The bond strength of the honeycomb at the joints meets the required standards, so no cracking is observed. The stress–strain curves of the quasi-static crushing experiment and LVI process simulation of the honeycomb are illustrated in Figure 13, and the agreement between the two is evident in both linear and nonlinear regions. The experimental results of the quasi-static crushing further validate the effectiveness of the LVI simulation model.

#### 4.1.2. The Effect of the Number of Plies

**Figure 14:**
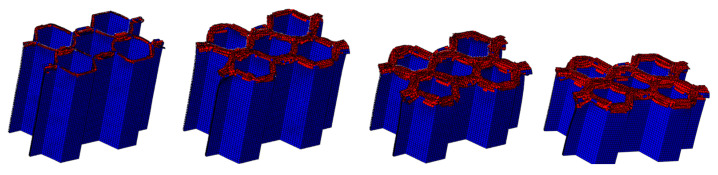
Crushing process of honeycomb with two plies.

**Figure 15:**
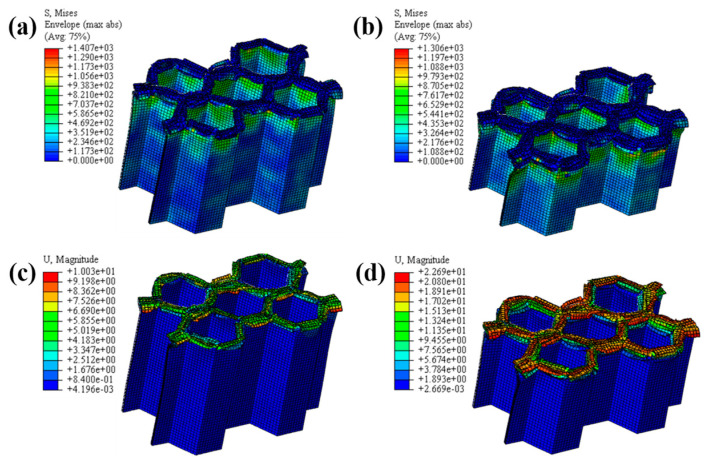
Honeycomb with two plies. Stress nephogram of (**a**) s = 12.5 mm and (**b**) s = 25 mm, and deformation nephogram of (**c**) s = 12.5 mm and (**d**) s = 25 mm.

**Figure 16:**
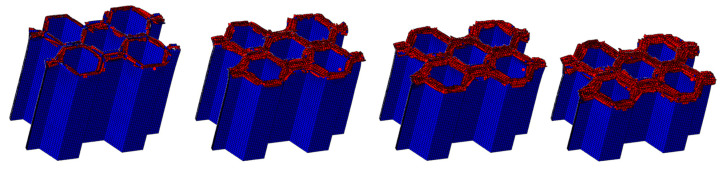
Crushing process of honeycomb with three plies.

**Figure 17:**
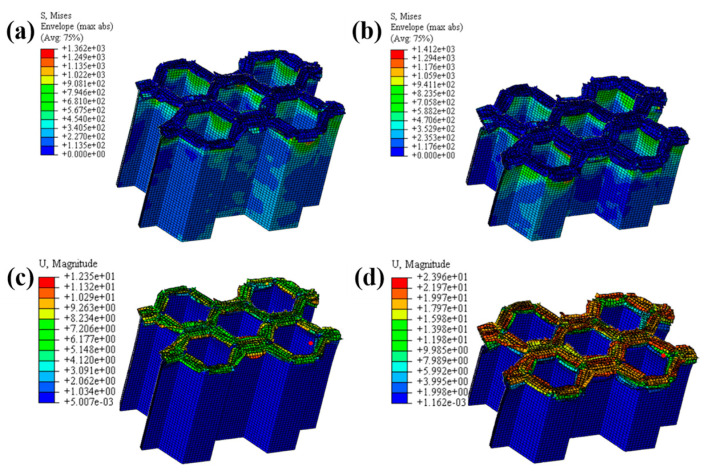
Honeycomb with three plies. Stress nephogram of (**a**) s = 12.5 mm and (**b**) s = 25 mm, and deformation nephogram of (**c**) s = 12.5 mm and (**d**) s = 25 mm.

The LVI collapse morphology of the honeycomb with two plies is shown in Figure 14 and Figure 15, and the LVI collapse morphology of the honeycomb with three plies is shown in Figure 16 and Figure 17. From these results, the out-flap and in-flap phenomena and the delamination characteristics of the laminate can be clearly seen. The stiffness of the honeycomb structure combined with a two-ply CFRP corrugated sheet structure is relatively low, and the buckling phenomenon will occur during the impact. For the honeycomb with three-ply CFRP, the overall internal structural stiffness is high, and the buckling instability phenomenon did not occur during compression collapse. In summary, the stress and deformation characteristics of CFRP honeycomb with different numbers of plies are basically the same, which shows that the stress and displacement values in the contact area are larger, and the original stress and deformation away from the contact area are smaller, i.e., a progressive collapse phenomenon. The material is subjected to greater loads under impact conditions, and there is stress wave transmission within the material compared to the quasi-static condition, so there will be significant stresses in the part closer to the contact region. The strength and stiffness of the composite material increase as the number of laminates increases, allowing the honeycomb structure to better withstand loads during impact, reducing the possibility of deformation and damage.

#### 4.1.3. The Effect of Different Ply Angles

**Figure 18:**
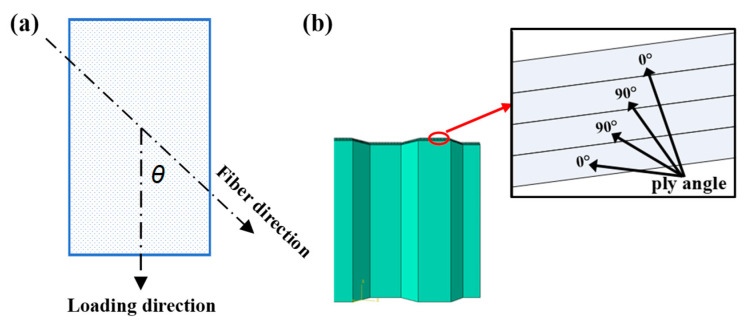
Ply angle in LVI experiment and FEM: (**a**) definition of ply angle, (**b**) initial ply angle formation [0°/90°]_s_.

Ply angle is an important factor affecting the energy absorption behavior of honeycomb structures. The laminates exhibit distinct mechanical properties depending on their layering angles, resulting in variations in both apparent elastic modulus and strength. As denoted in Figure 18a, ply angle *θ* represents the angle between the loading direction and the fiber direction. The mechanical properties of 0°-oriented laminates are usually tested, and the mechanical parameters of laminates with other ply angles can be converted and calculated using the stress rotation axis formula [34]. The honeycomb structure in this paper is formed through the alternating symmetrical layering of fibers oriented perpendicularly, and the initial ply angle formation is [0°/90°]_s_, as shown in Figure 18b.

**Figure 19:**
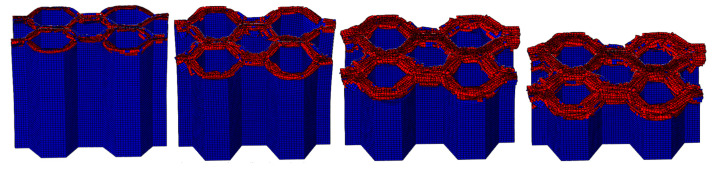
Crushing process of the honeycomb with [30°/−60°]_s_ plies.

**Figure 20:**
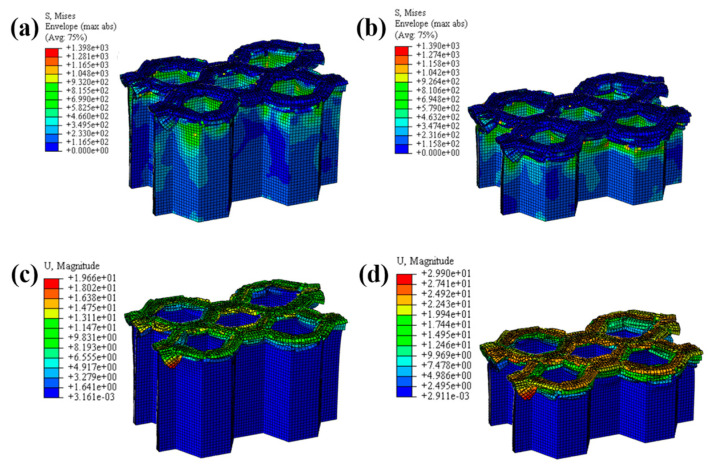
The honeycomb with [30°/−60°]_s_ plies. Stress nephogram of (**a**) s = 12.5 mm and (**b**) s = 25 mm, and deformation nephogram of (**c**) s = 12.5 mm and (**d**) s = 25 mm.

**Figure 21:**
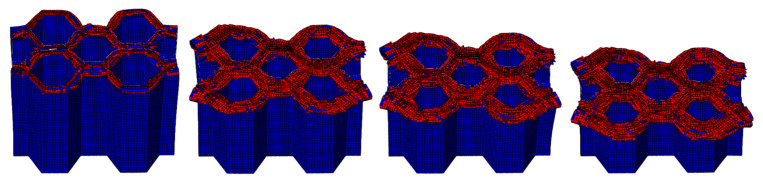
Crushing process of the honeycomb with [45°/−45°]_s_ plies.

**Figure 22:**
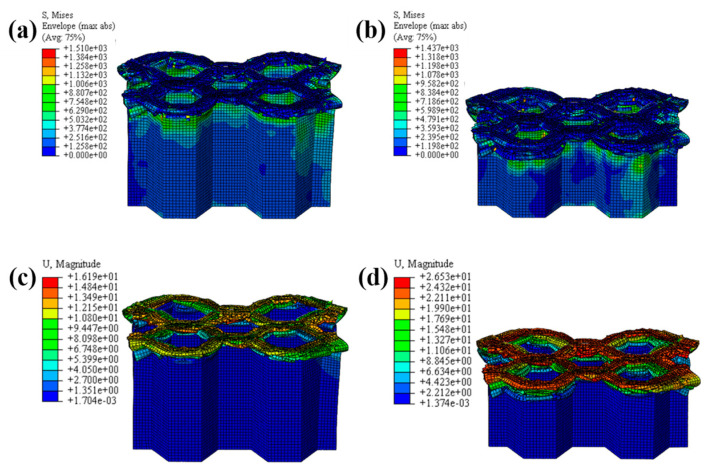
The honeycomb with [45°/−45°]_s_ plies. Stress nephogram of (**a**) s = 12.5 mm and (**b**) s = 25 mm, and deformation nephogram of (**c**) s = 12.5 mm and (**d**) s = 25 mm.

**Figure 23:**
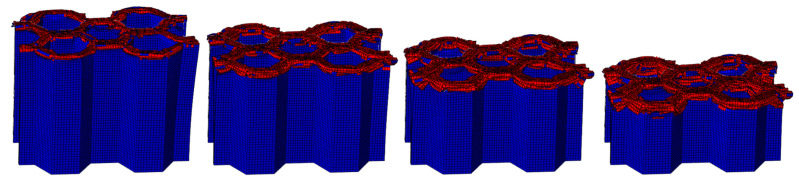
Crushing process of the honeycomb with [60°/−30°]_s_ plies.

**Figure 24:**
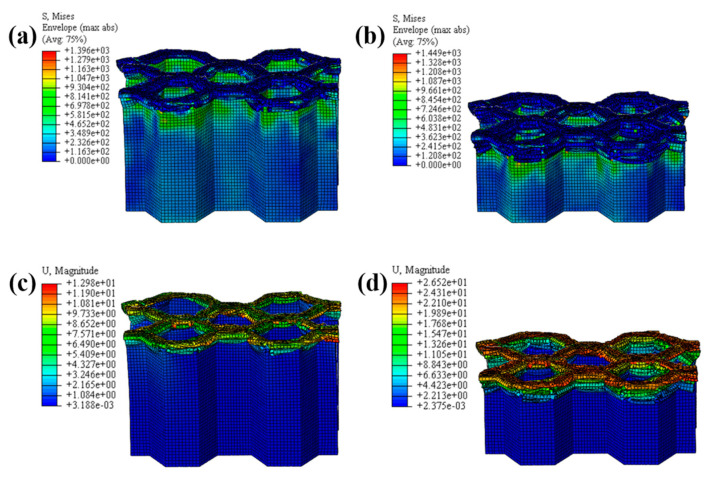
The honeycomb with [60°/−30°]_s_ plies. Stress nephogram of (**a**) s = 12.5 mm and (**b**) s = 25 mm, and deformation nephogram of (**c**) s = 12.5 mm and (**d**) s = 25 mm.

**Figure 25:**
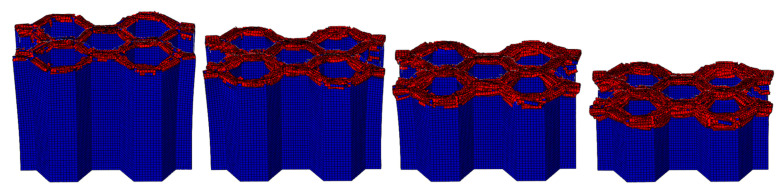
Crushing process of the honeycomb with [90°/−0°]_s_ plies.

**Figure 26:**
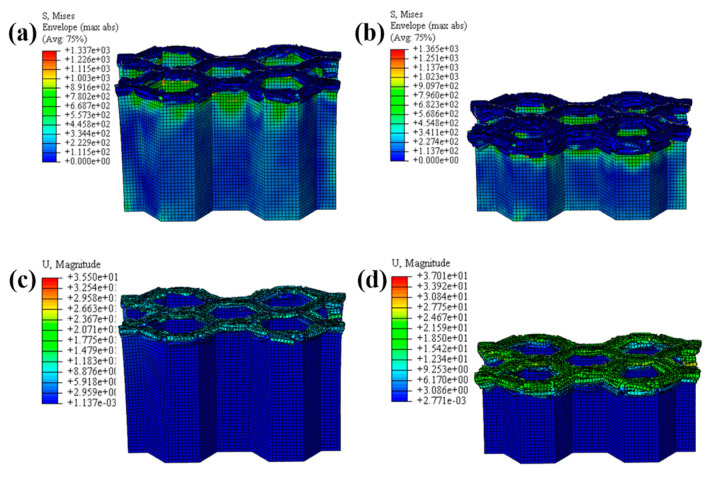
The honeycomb with [90°/−0°]_s_ plies. Stress nephogram of (**a**) s = 12.5 mm and (**b**) s = 25 mm, and deformation nephogram of (**c**) s = 12.5 mm and (**d**) s = 25 mm.

The crushing process of the honeycomb with [30°/−60°]_s_ plies is shown in Figure 19 and Figure 20. A small part of the honeycomb with this ply angle does not collapse and fails when compressed, and the failed part is tilted to both sides. The crushing process of the honeycomb with [45°/−45°]_s_ plies is shown in Figure 21 and Figure 22. Similarly, a small portion of the fiber matrix is not crushed to failure and collapses to the sides along with the failed crushed body. The CFRP honeycomb undergoes significant progressive failure and is continuously crushed. The crushing process of the honeycomb with [60°/−30°]_s_ plies is shown in Figure 23 and Figure 24. The crushing process of the honeycomb with [90°/0°]_s_ plies is shown in Figure 25 and Figure 26. The fiber of the outermost layer in this honeycomb structure is oriented perpendicular to the compression direction, making it susceptible to crushing on both sides upon impact. Consequently, its load-bearing capacity is relatively low, and its impact resistance is compromised.

The LVI collapse morphology and damage shapes of honeycombs with five different ply angles, namely [0°/90°]_s_, [30°/−60°]_s_, [45°/−45°]_s_, [60°/−30°]_s_, and [90°/0°]_s_, are basically the same. The red damage part of the collapse failure diagrams exhibits a curled morphology and turns to both sides. However, different ply angles will cause the CFRP honeycomb structures exhibit varying mechanical properties under LVI, resulting in different energy absorption capabilities.

### 4.2. Comparison of Energy Absorption

#### 4.2.1. Energy Absorption and Specific Energy Absorption

Energy absorption (EA) and specific energy absorption (SEA) are important indicators for evaluating the ability of a material to absorb energy when subjected to an external load. EA refers to the ability of a material to effectively absorb and dissipate energy when subjected to external impacts or loads, while SEA refers to the amount of energy that can be absorbed per unit volume or mass of a material during the process of energy absorption. The level of EA capacity and SEA is usually closely related to the composition, structural design, mechanical properties, and other factors of the material. The design of honeycomb structures, foam structures, porous structures, etc., can effectively disperse the impact force and increase the EA surface area, thus improving the EA effect. The EA is expressed as

(4) $$EA=\int_{0}^{l}Pdl$$

where P is the load carried by the honeycomb structure and l is the loading stroke.

Thereby, the SEA can be expressed as

(5) $$SEA=\frac{EA}{M}$$

where M is the mass of the honeycomb.

#### 4.2.2. Energy Absorption Effect of Dynamic Shock and Quasi-Static Compression

**Figure 27:**
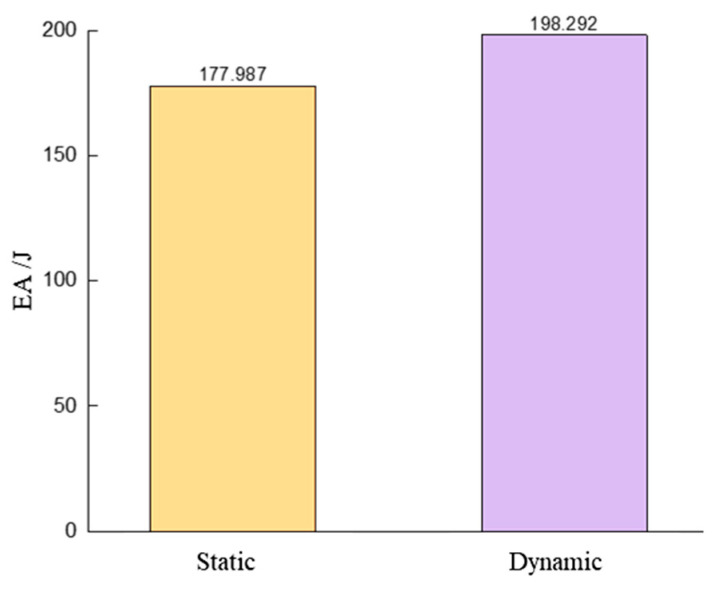
Comparison of EA in dynamic LVI and quasi-static compression.

As shown in Figure 27, the CFRP honeycomb exhibits better EA under dynamic impact loading conditions, which is an improvement of 11.41% compared to quasi-static compression. Under dynamic impact loading conditions, the action time is shorter, the load application rate is higher, and the strain rate increases. Due to the high strength and stiffness of CFRP, it can rapidly withstand the impact load in a short period of time and exhibits its excellent mechanical properties, thus achieving a better EA effect. Additionally, due to the presence of more energy dissipation mechanisms within the honeycomb, such as fiber damage, interfacial shear, and interaction of failed layer clusters, these mechanisms aid in efficiently absorbing and dispersing the impact energy, further enhancing the EA effect.

**Figure 28:**
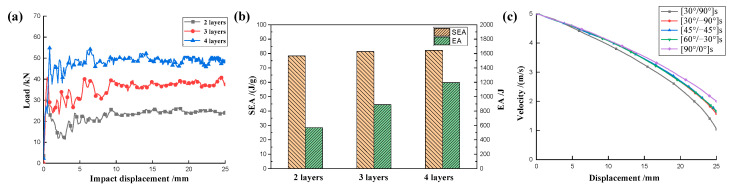
(**a**) Crushing load for the honeycomb with different layers, (**b**) EA and SEA relationship for the honeycomb with different layers, and (**c**) deceleration effect for the honeycomb with different ply angles.

As shown in Figure 28a, for the CFRP honeycomb structure, the number of fiber layers in the composite increases with the increase in the number of plies, which improves the strength and stiffness of the material and thus allows for more load sharing and better EA during impacts. However, although increasing the number of plies improves the strength and EA properties of the honeycomb, it does not significantly improve the SEA effect because, to some extent, increasing the number of plies, while increasing the strength, also increases the weight of the material, so that the amount of EA per unit of mass does not improve significantly but remains basically the same, as shown in Figure 28b. Therefore, when designing CFRP honeycombs, it is necessary to balance the relationship between the number of plies (stiffness, strength) and the EA and SEA effects, and choose the appropriate number of plies to meet the practical requirements. Since the boundary conditions of the rigid plate impacting the honeycomb with five different ply angles are the same and the geometric parameters are also the same, taking the velocity at 25 mm displacement of the rigid plate as the final velocity, the velocity change process can be obtained, as shown in Figure 28c. It can be seen that the fastest decrease in rigid plate velocity was observed for the honeycomb with a [0°/90°]_s_ ply angle, the decrease in rigid plate velocity was similar for [30°/−60°]_s_, [45°/−45°]_s_ and [60°/−30°]_s_, and the least decrease in rigid plate velocity was observed for the [90°/0°]_s_ ply configuration.

Here, the velocity drop ratio φ is defined to characterize the impact resistance and EA properties of the honeycomb with five different ply angles, i.e.,

(6) $$\varphi =\frac{V_{0}-V_{1}}{V_{0}}$$

where $V_{0}$ is the initial velocity of the rigid plate and $V_{1}$ is the final velocity of the rigid plate.

The larger value of φ indicates the better impact resistance of the CFRP honeycomb structure. After calculation, the velocity drop ratios for the five different ply angles are $\varphi_{A}=79.12\%$ ([0°/90°]_s_), $\varphi_{B}=68.49\%$ ([30°/−60°]_s_), $\varphi_{C}=66.88\%$ ([45°/−45°]_s_), $\varphi_{D}=66.86\%$ ([60°/−30°]_s_), and $\varphi_{E}=60.02\%$ ([90°/0°]_s_). As expected, the CFRP honeycomb with [0°/90°]_s_ ply angle exhibits the best impact resistance, while those with other ply angles show relatively worse performance. In practical applications, where impact load can be from any angle, the study of other ply angles is equally important, providing further reference and a basis for further research on the impact EA of CFRP honeycombs.

## 5. Conclusions

The present study employed CFRP prepreg as the primary material and utilized an autoclave forming process to fabricate CFRP corrugated sheet along with its composite counterpart, namely honeycomb. The compression and collapse behaviors of CFRP corrugated sheet and honeycomb were studied through quasi-static and LVI experiments. The finite element method was used for numerical simulation, and the computed results were extracted and compared with the experimental findings. The obtained results exhibited excellent agreement, thereby validating the accuracy of the simulation model and establishing a solid foundation for subsequent investigation pertaining to honeycomb structures. The following conclusions were drawn:

(1) During the crushing process, both the CFRP corrugated sheet and honeycomb are stably damaged, including matrix fracture, fiber fracture, matrix fragmentation, and layer failure. The crushed body flips from the center to the sides, while the crushed matrix and fiber flip to the outside and inside of the honeycomb holes, respectively.
(2) The well-established FEM demonstrates remarkable accuracy in various simulations, including quasi-static compression of CFRP corrugated sheet, LVI of CFRP corrugated sheet, and LVI of CFRP honeycomb. The validated model can be applied to the structural design of spacecraft, significantly reducing the design cycle and R&D expenses.
(3) A kind of index for evaluating the impact resistance was proposed: the velocity drop ratio. Under this evaluation index, the larger the value of the velocity drop ratio, the more effectively the CFRP honeycomb structure can slow down the velocity when subjected to impact, indicating that its impact resistance is better.
(4) The best impact resistance performance of the honeycomb structure was determined by numerical simulations using a ply angle of [0°/90°]_s_. The energy absorption performance increases with the number of layers, and the relationship between the two is approximately linear.
(5) A large amount of space inside the honeycomb structure can be used for enhancing mechanical properties. In subsequent research, there are some directions, such as the behavior of the CFRP honeycomb structure in high-speed impact, honeycomb filled with lightweight materials such as aluminum foam, polymer foam, and others, the optimal structure, and the mixture of materials and structures.

## Figures and Tables

**Table 1:** Material properties of CFRP prepreg.

Mechanical Properties	Symbol	Value
Longitudinal modulus (GPa)	*E*_11_	55.9
Transverse modulus (GPa)	*E*_22_ = *E*_33_	54.4
Shear modulus (GPa)	*G*_12_ = *G*_23_ = *G*_31_	4.2
Principal Poisson’s ratio	*ν*_12_	0.042
Longitudinal tensile strength (MPa)	*X_T_*	911.3
Longitudinal compressive strength (MPa)	*X_C_*	704.0
Transverse tensile strength (MPa)	*Y_T_*	770.1
Transverse compressive strength (MPa)	*Y_C_*	698.2
In-plane shear strength (MPa)	*S_L_*	131.6

**Table 2:** Damage parameters of CFRP [11].

Mechanical Properties	Symbol	Value
Fiber tensile fracture energy (kJ/m^2^)	*G_ft_*	125
Fiber compression fracture energy (kJ/m^2^)	*G_fc_*	250
Matrix tensile fracture energy (kJ/m^2^)	*G_mt_*	95
Matrix compression fracture energy(kJ/m^2^)	*G_mc_*	245
Type I fracture toughness (kJ/m^2^)	*G_Ic_*	0.504
Type II fracture toughness (kJ/m^2^)	*G_IIc_*	1.566
Normal nominal maximum stress (MPa)	*N*	54
Tangential nominal maximum stress (MPa)	*S* = *T*	70

**Table 3:** Strain-based element deletion definition.

Element Deletion Definition	Value
Maximum tensile strain in fiber direction	0.1
Maximum compression strain in fiber direction	0.1
Maximum tensile strain in the vertical fiber direction	0.1
Maximum compression strain in the vertical fiber direction	0.1
Maximum shear strain	0.3

## Data Availability

The original contributions presented in the study are included in the article, further inquiries can be directed to the corresponding authors.
